# Supplementary material for: An explainability framework for deep learning on chemical reactions exemplified by enzyme-catalysed reaction classification
Source: J Cheminform. 2023 Nov 23;15:113. doi: 10.1186/s13321-023-00784-y (PMC10668483; doi:10.1186/s13321-023-00784-y)
Supplement: Supplementary file 1 — Additional file 1: Fig. S1. Training and validation losses for the modelspresented in this work. Early stopping is implemented bymonitoring the mean validation loss of the 5 most recentepochs. The training is stopped if the improvement of thecurrent loss drops below 0.001. Fig. S2. Boxplots showing the distribution of accuraciesamong classes, subclasses, and sub-subclasses. The filledorange circle represents the mean. Fig. S3. Scatter plots showing the dependence of accuracyon sample size for models trained on Rhea data. Fig. S4. Scatter plots showing the dependence of accuracyon sample size for models trained on ECREACT data. [file 13321_2023_784_MOESM1_ESM.pdf]

Appendix A Supplementary Figures and Tables

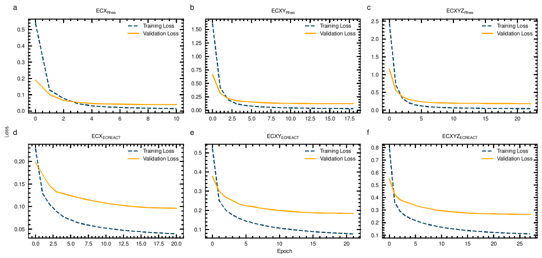

Fig. S1 Training and validation losses for the models presented in this work. Early stopping is implemented by monitoring the mean validation loss of the 5 most recent epochs. The training is stopped if the improvement of the current loss drops below 0.001.

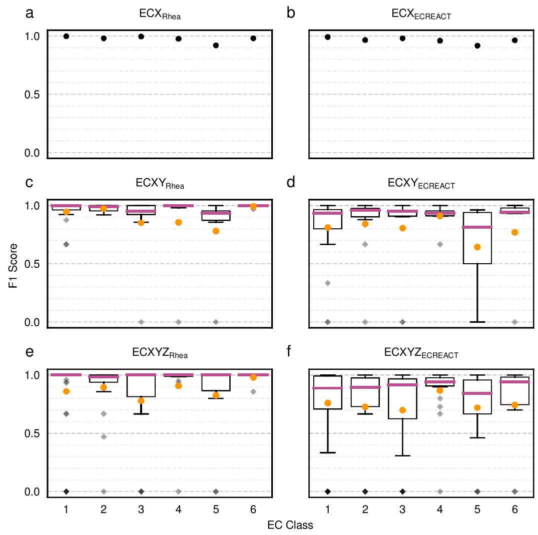

Fig. S2 Boxplots showing the distribution of accuracies among classes, subclasses, and sub-subclasses. The filled orange circle represents the mean.

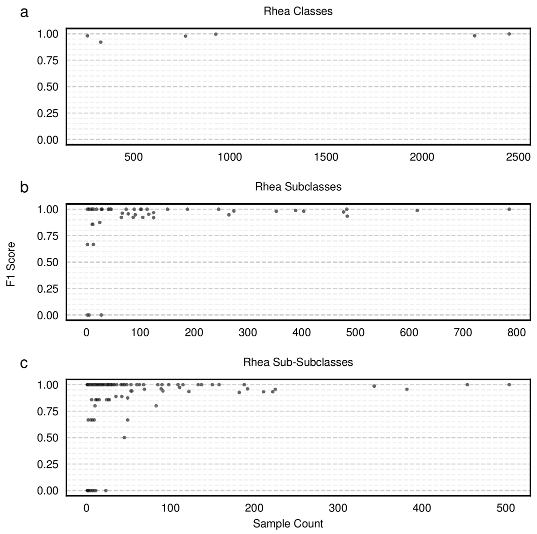

Fig. S3 Scatter plots showing the dependence of accuracy on sample size for models trained on Rhea data.

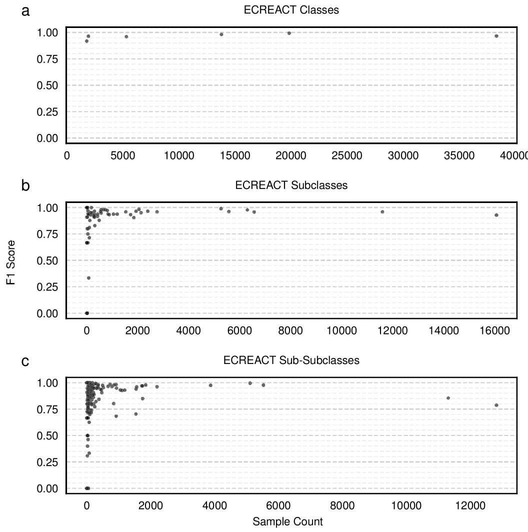

Fig. S4 Scatter plots showing the dependence of accuracy on sample size for models trained on ECREACT data.
